# Novel expression profiles of microRNAs suggest that specific miRNAs regulate gene expression for the sexual maturation of female *Schistosoma japonicum* after pairing

**DOI:** 10.1186/1756-3305-7-177

**Published:** 2014-04-10

**Authors:** Jun Sun, Suwen Wang, Chen Li, Yijiu Ren, Jinqiang Wang

**Affiliations:** 1 Institute for Infectious Diseases and Vaccine Development, Tongji University School of Medicine, Shanghai 200092, People’s Republic of China

**Keywords:** Schistosoma japonicum, Pairing, Solexa, miRNA, bantam, miRNA-1, miRNA-71, miRNA-7, miR-7-5p

## Abstract

**Background:**

*Schistosoma japonicum* is one of the major causative agents of schistosomiasis. The pairing of males and females leads to female sexual maturation and maintains this mature state. However, the mechanisms by which pairing facilitates sexual maturation are yet to be investigated.

**Methods:**

Parasites isolated from single- and double-sex cercariae-infected mice were analyzed by Solexa to uncover pair-regulated miRNA profiles. To reveal the biological functions of differentially expressed miRNAs among the samples, we predicted the target genes of these differentially expressed miRNAs and compared the gene expression between 23-d-old female schistosomula from double-sex infections (23DSI) and 23-d-old female schistosomula from single-sex infections (23SSI) by analyzing digital gene expression profiling (DGE). KEGG pathway analysis was used to investigate the relevant biological processes of these target genes to understand the significance of differentially expressed miRNAs after pairing.

**Results:**

The differentially expressed miRNA profiles of female 18- and 23-d post-single- and double-sex infections were analysed by Solexa. Similar miRNA profiles were observed in 18SSI and 18DSI, with the presence of identically expressed high-abundance miRNA, such as miRNA-1, miRNA-71b-5p and let-7. By contrast, in 23DSI and 23SSI, most of these high-abundance miRNAs were down-regulated. Furthermore, among all samples, bantam was distinctly up-regulated in 23 DSI, and miR-1, miR-71, miR-7-5p, and miR-7 were distinctly up-regulated in 23SSI. The transcriptomes of 23DSI and 23SSI revealed that the predicted target genes of miRNA-1, miRNA-71, miRNA-7, and miR-7-5p were associated with the ribonucleoprotein complex assembly and microtubule-based process. Conversely, the predicted target genes of bantam were related to the embryo development, development of primary sexual characteristics and regulation of transcription. KEGG pathway analysis revealed that in unpaired females, the highly-expressed miRNA-1, miRNA-71, miRNA-7, and miR-7-5p only inhibited the limited pathways, such as proteasome and ribosome assembly. Meanwhile, in paired mature females, highly-expressed bantam inhibited more biological pathways, such as the citrate cycle, glycolysis, fatty acid biosynthesis and RNA degradation.

**Conclusions:**

The differentially expressed miRNAs between 23SSI and 23DSI and their different functions indicated that more genes or metabolic pathways in paired mature females were inhibited than those in unpaired ones. The results suggested that after pairing, specific miRNAs regulated gene expression to lead to female sexual maturation.

## Background

Schistosomiasis is a chronic and debilitating parasitic disease caused by blood flukes of the genus *Schistosoma*. Over 200 million people are infected, and close to 800 million are at risk of this parasitic disease [1]. No successful vaccine is available for this disease. Praziquantel is the only drug used to treat schistosomiasis but is ineffective against young worms. Moreover, the drug resistance of the blood flukes to praziquantel has been reported [2]. Thus, identifying novel drug targets and developing alternative therapeutic strategies are urgent and necessary. In the hosts, males and females pair and eventually develop into adult worms, accompanied by remarkable morphological and molecular changes throughout their life cycle [3-7]. Previous studies have proposed that male contact is necessary for ovary and vitelline gland development. Moreover, this interaction is ultimately linked to the sexual maturation and maintenance of the mature state of females [8-10]. In evolutionary terms, schistosomes are likely to have evolved from hermaphroditic flukes to dioecious trematodes with an increasingly complete functional separation of the sexes. During the process, females still need the constant pairing contact with males to reach sexual maturation. To date, the exact mechanisms by which males influence female maturation are still unknown. Different factors have been suggested to be involved in schistosome sexual maturation, including physical or tactile contact [8,11], nutrition [12,13], and chemical stimuli [14,15]. However, the mechanisms by which pairing facilitates female development are yet to be investigated.

MicroRNAs (miRNAs) are a class of small non-coding RNAs (18–25 nucleotides long) generated from endogenous transcripts that form hairpins [16], functioning in transcriptional and post-transcriptional regulation of gene expression [17,18]. The binding of miRNA to a target mRNA typically results in gene silencing through translational repression and target mRNA degradation [19]. Recently, miRNAs have been reported to be involved in translational activation [20] and heterochromatin formation [21]. Through these actions, miRNAs regulate gene expression during development, differentiation, proliferation, death, and metabolism in many organisms [22]. Recently, some schistosome miRNAs have been identified [23-29], which provided some insight into the role of miRNAs in schistosome development.

During the development of *S. japonicum*, males and females begin to pair about 18 d post-infection, and the female begins to lay eggs about 24 d post-infection [30]. The role miRNAs play after pairing remains unknown. In this study, the expression profile of miRNAs of *S. japonicum* before and after pairing were investigated. Moreover, based on the analysis of their predicted targets, the different and specific functional requirements before and after pairing will be determined based on the novel profiles of miRNAs.

## Methods

### Ethics statement

This study was carried out in strict accordance with the recommendations of the Regulations for the Administration of Affairs Concerning Experimental Animals of the State Science and Technology Commission. The protocol was approved by the Internal Review Board of Tongji University School of Medicine.

### Unisexual and paired infections

*Oncomelania hupensis* snails were obtained from the Jiangsu Institute of Schistosome Diseases, Jiangsu Province, China. To obtain single-sex female worms, the snails were exposed to a single miracidium, which was generated from eggs acquired from the liver of infected rabbits or mice. Approximately 100 to 150 freshly shed cercariae were used to percutaneously infect each mouse. Schistosomula were recovered by perfusion within 18 d and 23 d post-infections. The worms were washed in cold saline solution and checked by microscopy for possible undesirable mixed-sex infections. We separated single-sex female worms and froze them at −80°C until further processing of the samples.

To obtain double-sex female worms, about 100 to 150 multiple cercariae freshly shed by snails were used to percutaneously infect each mouse. The mice were sacrificed 18 d and 23 d post-infection, respectively. Females were recovered by washing with cold saline solution. The 23-d-old females were carefully separated from the paired worms under a microscope. All samples were frozen until further processing.

### RNA extraction and small RNA library construction and sequencing

Total RNA was extracted using Trizol reagent (Invitrogen Life Technologies) according to the manufacturer’s instructions. RNA concentration and purity were evaluated spectrophotometrically at 260 nm and 280 nm, respectively, using a NanoDrop ND1000 spectrophotometer and an Agilent 2100 Bioanalyzer (Agient Technologics, Palo Alto, CA). RNA samples were stored at −80°C.

The construction of small RNA libraries was carried out as described in the Additional file 1. Briefly, RNAs with sizes ranging from 18–30 nucleotides (nt) were excised and purified and ligated to 3′ and 5′ adaptors, and further converted into 62–75 nt single-stranded cDNAs. The cDNAs were amplified using Illumina’s 3′ adaptor reverse primer and 5′ adaptor forward primers. The purified PCR products were sequenced by an Illumina Genome Analyzer at the BGI (Beijing Genomics Institute, Shenzhen, China).

### Mapping sequence reads to the reference genome

All raw datasets produced by deep sequencing from the libraries (23DSI, 23SSI, 18DSI, and 18SSI) were analysed as follows. Clean reads were obtained after removal of the low quality reads, insert null reads, adaptor null reads, reads with ployA tail, and reads shorter than 18 nt. The small RNAs with sizes ranging from 18 nt to 30 nt were used for further analyses. All identical sequences were counted and merged as unique sequences, herein referred to as sequence tags. The unique reads along with their associated read counts were mapped to the *S. japonicum* genome sequences (http://www.chgc.sh.cn/japonicum/Resources.html) using the programme SOAP [31]. The small RNAs which matched with known rRNAs, tRNAs, small nuclear RNAs (snRNAs), and small nucleolar RNAs (snoRNAs) deposited in the Rfam database (ftp://selab.janelia.org/pub/Rfam/) and NCBI GenBank (http://www.ncbi.nlm.nih.gov/GenBank) were excluded. All unique sequences were utilised for the BLASTN search against the miRNA database (miRBase 18.0) (http://www.mirbase.org/) to identify conserved miRNAs (Additional file 1).

### Bioinformatic analysis of *S. japonicum* small RNAs

Small RNA tags were aligned to the miRNA precursor/mature miRNA of corresponding species in miRBase18. Detailed information of the alignment, including the structure of known miRNA precursor, and length and count of tags from the sample, among others, were collected. To make every unique small RNA mapped to only one annotation, we followed the following priority rule: rRNA etc. (in which Genbank > Rfam) > known miRNA > repeat > exon > intron3. The total rRNA proportion should be less than 40%. The expression of the miRNAs in four samples were visualised by plotting the Log2-ratio figure and the scatter plot. The procedures adopted were as follows: (1) the expression of miRNA in two samples (control and treatment) were normalised to get the expression in transcript per million (TPM) with the normalisation formula (normalised expression = actual miRNA count/total count of clean reads × 1000000); and (2) the fold-change and P-value were calculated from the normalised expression, and log2ratio and scatter plots were generated using the fold-change formula (fold change = log2 (treatment/control)).

To understand the molecular function of differentially expressed miRNAs, we used the algorithms PicTar [32] and TargetScan [33] to predict their target mRNAs. All predicted target genes were evaluated by the scoring system and the criteria described by Chi *et al*. [34]. Sequences with total scores less than 3.0 points were considered as miRNA potential targets. Based on the digital gene expression profiling (DGE) analysis and negative correlation analysis between miRNA profiles and genes expression profiles, the false positive miRNA targets were further excluded.

### Identification of differentially expressed genes and pathways in 23DSI and 23SSI

DGE was used to analyse the whole genome gene expression in 23DSI and 23SSI. Raw reads were filtered to obtain high quality data in the Tag-seq libraries of 23DSI and 23SSI. All of the clean tags were mapped to the *S. japonicum* genome (http://www.chgc.sh.cn/japonicum/Resources.html) (predicted coding genes). The clean tags mapped to the reference sequences from multiple genes were filtered, and the remaining clean tags were designed and annotated as unambiguous clean tags. The initial counts of the clean tags of each gene were normalised (transcripts per million) to obtain the normalised gene expression [35,36]. All differentially expressed genes were mapped to terms in the GO database, to identify significantly enriched GO terms in DEGs compared with the genome background. Pathway enrichment analysis was used to identify significantly enriched metabolic pathways or signal transduction pathways in DEGs compared with the whole genome background. Detailed pathway information was determined with the KEGG database. More details are shown in Additional file 2. The regulatory effect of differentially expressed miRNA in worms after pairing were predicted by analysing the functions of their predicted target genes and by comparing the proportion of their predicted target genes in differentially expressed genes.

### MiRNA quantification by quantitative RT-PCR analysis

To ensure specificity of the PCR amplification of the cDNAs, we designed the primers based on the following criteria: predicted melting temperatures of 66 ± 2°C, limited self-complementarity, and primer length ranging from 20–22 nt. The first cDNA strand was synthesised from 0.15 μg total RNA using PrimeScript® RT reagent Kit (Perfect Real Time) (Takara Code: DRR037A). Stem-loop qRT-PCR was performed to quantify the sex-biased expressed miRNAs. Stem-loop RT primers were designed to reverse-transcribe the target miRNAs into cDNAs using total RNAs isolated from female worms (from the same samples used for Solexa sequencing). The 20 μl reaction RT system contained 2 μl of total RNA (0.15 μg), 2 μl (1 μM) of each individual stem-loop RT primer, 4 μl 5× PrimeScript® Buffer (for Real Time), 1 μl PrimeScript® RT Enzyme Mix I, and 11 μl RNase Free dH_2_O.

The following were designed as the RT primers of the miRNAs: Sja-mir-71 5′ GTCGTATCCAGTGCAGGGTCCGAGGTATTCGCACTGGATACGACCTCACTAC3′; Sja-bantam 5′GTCGTATCCAGTGCAGGGTCCGAGGTATTCGCACTGGATACGACACCAGCTT3′; and Sja-mir-1 5′ GTCGTATCCAGTGCAGGGTCCGAGGTATTCGCACTGGATACGACTTCATACC3′. The primer 5′ TATGGAACGCTTCACGATTTTG3′ was designed for the RT of U6. The cDNAs were synthesised by incubation for 30 min at 16°C, 30 min at 42°C, and 15 min at 70°C. The products were amplified using SYBR® Premix Ex Taq™ (Tli RNaseH Plus) (Takara Code: RR420A) in an ABI Prism 7300 sequence detection system (Applied Biosystems) according to the manufacturer’s instruction. Our system contained 2 μl of cDNA from the RT reaction product (1:50 dilution), 5 μl of 2× SYBR Premix Ex Taq™ (TaKaRa), and 0.4 μl of 10 μM forward and reverse primers. The following were designed as the forward primers of the miRNAs: Sja-mir-71 5′ATGGTTCGTGGGTGAAAGACGATGGT3′; Sja-mir-1 5′ ATGGTTCGTGGGTGGAATGTAAAGAAGTATGG3′; and Sja-bantam 5′ ATGGTTCGTGGGTGAGATCGCGATTAAA3′. The primer 5′GCAGGGTCCGAGGTATTC3′ was used as the common reverse primer. The forward primer 5′CGGCGGTACATATACTAAAAT3′ and reverse primer 5′AACGCTTCACGATTTTGCGTA3′ were used to amplify the U6 gene as an endogenous control within each sample. Relative levels of gene expression were calculated using the 2^-ΔΔCt^ method. Each sample was analysed for primer dimer, contamination, or mispriming by inspection of their dissociation curves.

### Statistical analysis

Results were presented as mean ± standard deviation from at least three independent experiments. Statistical analyses were performed using one-way ANOVA and Student’s t-test. A value of *P* < 0.05 was considered statistically significant.

## Results

### Screening of miRNAs in *S. japonicum* from both single- and double-sex female worms at 18 d and 23 d post-infection

**Figure 1:**
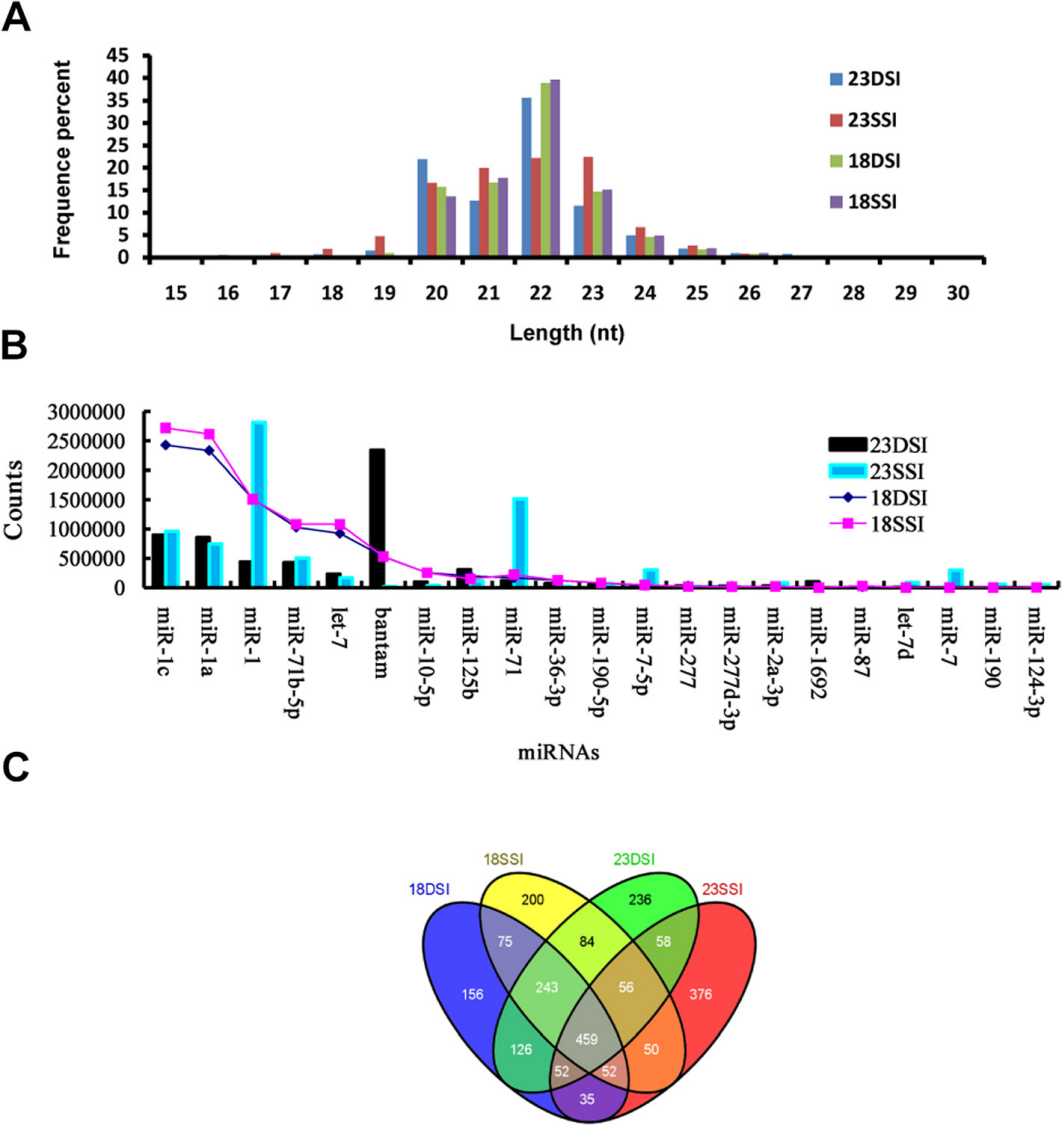
**Comparison of the miRNA profiles from single- and double-sex female worms at 18 d and 23 d post-infection. A.** Length, distribution, and abundance of small RNA tags of 18DSI, 18SSI, 23DSI, and 23SSI. **B.** miRNA profiles of 23DSI and 23SSI, in comparison with those of 18DSI and 18SSI. Different characteristics are shown among four profiles. **C.** The quantity of exclusive and common miRNAs among different samples. The Venn diagram is created with an online Venn diagram maker. (http://bioinfogp.cnb.csic.es/tools/venny/index.html)

To understand the effect of pairing on the development of female *S. japonicum*, we constructed cDNA libraries derived from 18–30 nt long RNAs isolated from both single- and double-sex female worms at 18 and 23 d post-infection. We sequenced the RNAs using an Illumina (Solexa) DNA sequencer, yielding a total of 14 333 070, 14 425 899, 14 624 020, and 12 268 947 clean reads corresponding to 98.92%, 97.27%, 99.07%, and 97.82% of high-quality reads for 18-d-old female schistosomula from 18DSI, 23DSI, 18SSI, and 23SSI, respectively. The sequencing tags were merged, and the expression level of each unique tag was normalised to TPM as previously described [37-39]. Our results showed that the majority of miRNA were between 20 nt to 24 nt in long (Figure 1A).

### Differential profiles of miRNAs in female *Schistosoma japonicum* from both single- and double-sex female worms at 18 and 23 d post-infection

Similar miRNA profiles were observed between 18SSI and 18DSI (Figure 1B). They shared 829 commonly known miRNAs (Figure 1C). The miRNAs with high-abundance, such miRNA-1c, miRNA-1a, miRNA-1, miRNA-71b-5p, let-,7 and so on showed identical expression. By contrast, the levels of all of these high-abundance miRNAs were down-regulated in 23DSI and 23SSI compared with 18 DSI or 18SSI (Figure 1B). Only the amount of bantam in 23DSI was up-regulated far more than that in 18 DSI, 18SSI, or 23 SSI. Compared with 18SSI, 23DSI shared a little more known miRNA with 18DSI (Figure 1C). Similarly, 23SSI shared more known miRNA with 18SSI than with 18DSI. Similar characteristics were shared between the miRNA profiles of 23SSI and 23DSI. For example, their levels of miR-1c, miR-1a, miR-1, miRNA-71b-5p, and let-7 were far lower than those in 18 DSI or 18SSI. Moreover, they shared 625 commonly known miRNAs (Figure 1C). Almost half of the miRNAs of 23DSI and 23SSI were identical. However, the expression of a few miRNAs in the worms exhibited distinct differences. For example, the higher expression of bantam was observed only in 23DSI, whereas higher expression of miR-1, miR-71, miR-7-5p, and miR-7 manifested only in 23SSI (Figure 1B).

### Confirmation of differentially expressed miRNAs by quantitative RT–PCR analysis

**Figure 2:**
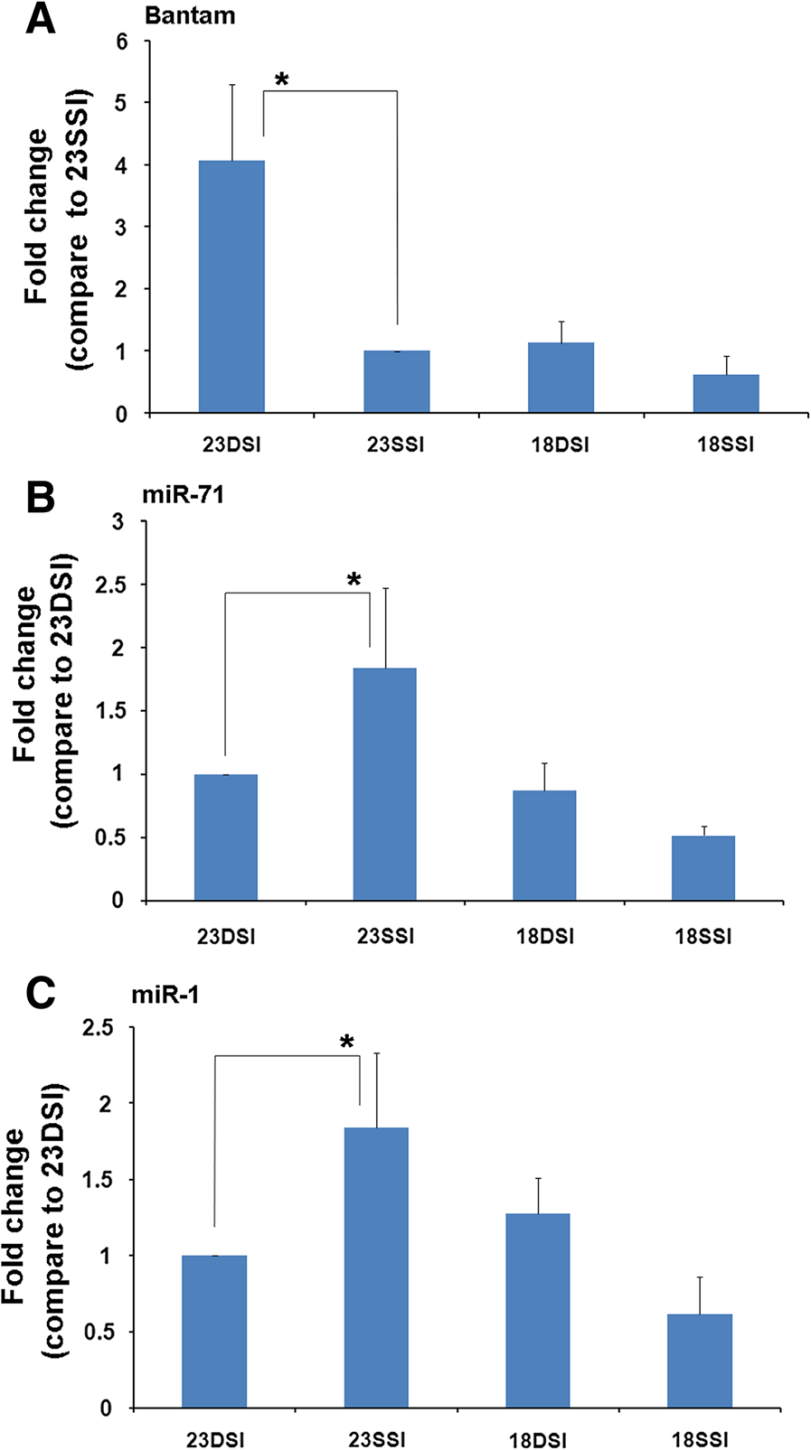
**Quantification of miRNAs dominantly expressed in 18DSI, 18SSi, 23DSI, and 23SSI. A.** Bantam, with respect to 23SSI, was significantly up-regulated in 23DSI (*P* < 0.01). **B.** miR-71, with respect to 23DSI, was significantly up-regulated in 23SSI (*P* < 0.05). **C.** miR-1, with respect to 23DSI, was significantly up-regulated in 23SSI (*P* < 0.01).

To confirm the differentially expressed miRNAs in 23DSI, 23SSI, 18DSI, and 18SSI, bantam, miRNA-1, and miR-71 were selected for quantitative RT–PCR analysis. The results of the RT-PCR showed that bantam was more abundant in 23DSI than in 23SSI, 18DSI, and 18SSI (Figure 2A). Similarly, higher amount of miR-71 (Figure 2B) and miR-1 (Figure 2C) were observed in 23SSI than in 23DSI. For the three miRNAs, quantitative RT–PCR analysis showed consistent expression with the Solexa analysis.

### Differential expression of the predicted target genes of bantam and miRNA-1-miRNA-71-miRNA-7-5p- miR-7 between samples from 23 DSI and 23SSI

To analyse the effect of the differential expression of miRNAs on female development after pairing, we sequenced the libraries of 23DSI and 23SSI, predicted the target genes of miRNA-1-miRNA-71-miRNA-7-miR-7-5p (Additional file 3: Table S1) and bantam (Additional file 4: Table S2), and analysed the differential expression of these genes in 23DSI compared with 23SSI. To reduce the false positives in the target gene prediction, we just analysed the predicted targets identified in the differentially expressed genes between 23DSI and 23SSI. Among the differentially expressed genes of 23DSI, we found that in the down-regulated genes of 23DSI, many predicted targets of bantam regulated protein metabolic process, mitotic spindle organisation, embryo development, glucose catabolic process, RNA metabolic process, apoptosis, hexose metabolic process, gene expression, mRNA transport, nematode larval development, multicellular organismal aging, development of primary sexual characteristics, translation, regulation of transcription, signalling, membrane lipid metabolic process, and the glucose metabolic process, among others (Table 1 and Additional file 5: Table S3). By contrast, in the up-regulated genes of 23DSI, the predicted target genes of miR-1-miR-71-miR-7-miR-7-5p appeared to regulate the ribonucleoprotein complex assembly, cellular protein complex assembly, microtubule-based process, response to oxidative stress, multicellular organismal aging, respiratory electron transport chain, pyrimidine ribonucleoside triphosphate biosynthetic process, positive regulation of epithelial cell differentiation, positive regulation of cell proliferation, apoptosis, energy coupled proton transport, electron transport chain, ATP synthesis-coupled proton transport, anatomical structure formation involved in morphogenesis, ribonucleoprotein complex biogenesis, mitotic cell cycle, larval development, microtubule polymerisation or depolymerisation, female gamete generation, regulation of transcription from RNA polymerase II promoter, and imaginal disc development, among others (Table 2 and Additional file 6: Table S4).

**Table 1:** Selected predicted target genes of bantam in the down-regulated genes in 23DSI

**Gene**	**RawIntensity-23SSI**	**RawIntensity-23DSI**	**log2 Ratio(23DSI/23SSI)**	**P-Value**	**FDR**	**GO Component**	**GO Function**	**GO Process**
Sjc_0013630|Sjp_0013630|SJC_S000059.548289	3987	208	−4.25	0	0	-	-	gi|257206034|emb|CAX82668.1|/1.09956e-27/Troponin T
Sjc_0007940|Sjp_0007940|SJC_S000025.694320	3316	400	−3.04	0	0	GO:0004175//endopeptidase activity	GO:0019222//regulation of metabolic process;GO:0019538//protein metabolic process	gi|30995341|gb|AAO59414.2|/0/cathepsin B endopeptidase
Sjc_0018120|Sjp_0018120|SJC_S000088.269899	3165	1412	−1.15	0	0	GO:0005488//binding;GO:0005198//structural molecule activity	GO:0007052//mitotic spindle organization;GO:0010467//gene expression	gi|226489394|emb|CAX75841.1|/1.27333e-22/Ribosomal protein L11
Sjc_0083460|Sjp_0083460|SJC_S001708.6042	2501	328	−2.92	0	0	GO:0016740//transferase activity	GO:0009987//cellular process;GO:0009790//embryo development	gi|308507601|ref|XP_003115984.1|/0/hypothetical protein CRE_08793
Sjc_0112990|Sjp_0112990|SJC_S007878.4178	1851	267	−2.78	0	0	GO:0004175//endopeptidase activity	GO:0019538//protein metabolic process	gi|56753605|gb|AAW25005.1|/3.77535e-176/SJCHGC02852 protein
Sjc_0031630|Sjp_0031630|SJC_S000203.86734	1450	109	−3.72	0	0	GO:0016832//aldehyde-lyase activity	GO:0006007//glucose catabolic process	gi|226475766|emb|CAX71973.1|/0/Aldolase
Sjc_0068090|Sjp_0068090|SJC_S000902.40949	1040	445	−1.21	0	0	GO:0016857//racemase and epimerase activity, acting on carbohydrates and derivatives;GO:0048037//cofactor binding	GO:0016070//RNA metabolic process;GO:0006915//apoptosis;GO:0019318//hexose metabolic process;GO:0010467//gene expression;GO:0051028//mRNA transport	gi|226487130|emb|CAX75430.1|/0/putative UDP-galactose-4-epimerase
Sjc_0038080|Sjp_0038080|SJC_S000276.119101	985	321	−1.6	0	0	GO:0016670//oxidoreductase activity, acting on a sulfur group of donors, oxygen as acceptor	GO:0008152//metabolic process	gi|76156276|gb|AAX27495.2|/0/SJCHGC06250 protein
Sjc_0006800|Sjp_0006800|SJC_S000021.979701	935	434	−1.09	0	0	GO:0004175//endopeptidase activity	GO:0019538//protein metabolic process	gi|226476888|emb|CAX72314.1|/0/cathepsin D (lysosomal aspartyl protease)
Sjc_0103990|Sjp_0103990|SJC_S004191.40055	664	260	−1.34	0	0	-	-	gi|256090158|ref|XP_002581079.1|/0/eukaryotic translation initiation factor 2c
Sjc_0044670|Sjp_0044670|SJC_S000376.73872	583	146	−1.98	0	0	GO:0005515//protein binding;GO:0000166//nucleotide binding	GO:0009790//embryo development;GO:0002119//nematode larval development;GO:0010259//multicellular organismal aging;GO:0045137//development of primary sexual characteristics	gi|226469288|emb|CAX70123.1|/1.05716e-112/heat shock protein 90 kDa alpha
Sjc_0092310|Sjp_0092310|SJC_S002488.15565	574	11	−5.69	0	0	GO:0005488//binding	GO:0006412//translation	gi|238658344|emb|CAZ29402.1|/0/expressed protein
Sjc_0060730|Sjp_0060730|SJC_S000689.17829	490	26	−4.22	0	0	GO:0015175//neutral amino acid transmembrane transporter activity;GO:0015291//secondary active transmembrane transporter activity	GO:0015804//neutral amino acid transport	gi|226468260|emb|CAX69807.1|/0/solute carrier family 6 (neurotransmitter transporter, glycine)
Sjc_0087030|Sjp_0087030|SJC_S001927.503	447	75	−2.56	0	0	GO:0005488//binding;GO:0016301//kinase activity	GO:0009987//cellular process	gi|226487670|emb|CAX74705.1|/0/putative Elongation factor 2 kinase (eEF-2 kinase)
Sjc_0053950|Sjp_0053950|SJC_S000536.83029	331	19	−4.11	0	0	GO:0005488//binding;GO:0003712//transcription cofactor activity	GO:0050896//response to stimulus;GO:0006355//regulation of transcription, DNA-dependent;GO:0023052//signaling	gi|56753834|gb|AAW25114.1|/9.32058e-50/SJCHGC01061 protein
Sjc_0094720|Sjp_0094720|SJC_S002677.1428	304	12	−4.65	0	0	-	GO:0006643//membrane lipid metabolic process	gi|56758970|gb|AAW27625.1|/0/SJCHGC01869 protein
Sjc_0101270|Sjp_0101270|SJC_S003878.693	100	12	−3.05	0	0	GO:0016868//intramolecular transferase activity, phosphotransferases	GO:0006006//glucose metabolic process	gi|257206128|emb|CAX82715.1|/2.08691e-122/putative phosphoglucomutase 2

**Table 2:** Selected predicted target genes of miR-1- miR-71- miR-7- miR-7-5p in the up-regulated genes in 23DSI

**Gene**	**RawIntensity-23SSI**	**RawIntensity-23DSI**	**log2 Ratio(23DSI/23SSI)**	**P-Value**	**FDR**	**GO Component**	**GO Function**	**GO Process**
Sjc_0070710|Sjp_0070710|SJC_S000983.72365	6928	14676	1.1	0	0	GO:0005198//structural molecule activity	GO:0006412//translation;GO:0022618//ribonucleoprotein complex assembly	gi|226475894|emb|CAX72037.1|/1.92699e-63/40S ribosomal protein S17
Sjc_0032370|Sjp_0032370|SJC_S000210.378287	1955	11833	2.61	0	0	GO:0016705//oxidoreductase activity, acting on paired donors, with incorporation or reduction of molecular oxygen;GO:0016209//antioxidant activity	GO:0008152//metabolic process;GO:0019725//cellular homeostasis	gi|226489432|emb|CAX75860.1|/2.80977e-109/Thioredoxin peroxidase
Sjc_0004280|Sjp_0004280|SJC_S000011.664038	4263	10833	1.36	0	0	GO:0017111//nucleoside-triphosphatase activity	GO:0043623//cellular protein complex assembly;GO:0009207;GO:0007017//microtubule-based process	gi|256087763|ref|XP_002580033.1|/0/alpha tubulin
Sjc_0066500|Sjp_0066500|SJC_S000837.212969	4057	10368	1.37	0	0	GO:0003676//nucleic acid binding;GO:0005198//structural molecule activity	GO:0010467//gene expression	gi|226475302|emb|CAX71939.1|/3.31945e-63/small subunit ribosomal protein S20e
Sjc_0054240|Sjp_0054240|SJC_S000544.55028	4128	8942	1.13	0	0	GO:0015078//hydrogen ion transmembrane transporter activity	GO:0006979//response to oxidative stress;GO:0010259//multicellular organismal aging;GO:0022904//respiratory electron transport chain;GO:0017062//respiratory chain complex III assembly	gi|29841122|gb|AAP06135.1|/2.29565e-72/SJCHGC00717 protein
Sjc_0009310|Sjp_0009310|SJC_S000031.859578	1125	6729	2.59	0	0	GO:0005198//structural molecule activity	GO:0010467//gene expression	gi|226478448|emb|CAX78482.1|/3.46982e-91/putative ribosomal protein L18a
Sjc_0058770|Sjp_0058770|SJC_S000642.39790	998	4195	2.08	0	0	GO:0032559;GO:0016776//phosphotransferase activity, phosphate group as acceptor	GO:0009209//pyrimidine ribonucleoside triphosphate biosynthetic process;GO:0006796//phosphate metabolic process;GO:0030858//positive regulation of epithelial cell differentiation;GO:0008284//positive regulation of cell proliferation;GO:0006915//apoptosis	gi|189502914|gb|ACE06838.1|/5.59719e-79/unknown
Sjc_0046710|Sjp_0046710|SJC_S000412.116878	1504	4011	1.43	0	0	-	-	gi|226472284|emb|CAX77178.1|/8.40244e-140/ribosomal protein L7a
Sjc_0075950|Sjp_0075950|SJC_S001243.77896	603	3843	2.68	0	0	GO:0003723//RNA binding;GO:0005198//structural molecule activity	GO:0010467//gene expression	gi|29841451|gb|AAP06483.1|/3.70409e-88/similar to NM_057813 ribosomal protein L9 in Ictalurus punctatus
Sjc_0091980|Sjp_0091980|SJC_S002467.11983	921	3113	1.77	0	0	GO:0017111//nucleoside-triphosphatase activity;GO:0032561	GO:0043623//cellular protein complex assembly;GO:0009207;GO:0007017//microtubule-based process	gi|226487270|emb|CAX75500.1|/0/Tubulin alpha-1 chain
Sjc_0059730|Sjp_0059730|SJC_S000664.27965	704	2801	2.01	0	0	GO:0019829//cation-transporting ATPase activity;GO:0015078//hydrogen ion transmembrane transporter activity;GO:0046872//metal ion binding;GO:0032559;GO:0043498//cell surface binding;GO:0015662//ATPase activity, coupled to transmembrane movement of ions, phosphorylative mechanism;GO:0004888//transmembrane receptor activity	GO:0015988//energy coupled proton transport, against electrochemical gradient;GO:0006929//substrate-dependent cell migration;GO:0022900//electron transport chain;GO:0015986//ATP synthesis coupled proton transport;GO:0006897//endocytosis;GO:0030641//regulation of cellular pH;GO:0048646//anatomical structure formation involved in morphogenesis;GO:0006839//mitochondrial transport	gi|226487050|emb|CAX75390.1|/0/ATP synthase, H + transporting, mitochondrial F1 complex, beta polypeptide
Sjc_0116280|Sjp_0116280|SJC_S008728.7876	857	2290	1.43	0	0	GO:0005198//structural molecule activity	GO:0022613//ribonucleoprotein complex biogenesis;GO:0006412//translation	gi|226469644|emb|CAX76652.1|/4.83022e-166/deoxyribonuclease
Sjc_0082530|Sjp_0082530|SJC_S001652.6464	659	1887	1.53	0	0	GO:0015662//ATPase activity, coupled to transmembrane movement of ions, phosphorylative mechanism;GO:0005515//protein binding;GO:0032559	GO:0000278//mitotic cell cycle;GO:0044267//cellular protein metabolic process;GO:0006909//phagocytosis	gi|56754732|gb|AAW25551.1|/0/SJCHGC06338 protein
Sjc_0071910|Sjp_0071910|SJC_S001024.53841	605	1823	1.6	0	0	GO:0017111//nucleoside-triphosphatase activity;GO:0032561	GO:0000278//mitotic cell cycle;GO:0035556//intracellular signal transduction;GO:0002164//larval development;GO:0031109//microtubule polymerization or depolymerization;GO:0007292//female gamete generation;GO:0051169//nuclear transport;GO:0015031//protein transport;GO:0009790//embryo development	gi|256085737|ref|XP_002579070.1|/2.37251e-121/ran
Sjc_0051340|Sjp_0051340|SJC_S000485.44512	235	1710	2.88	0	0	GO:0003677//DNA binding;GO:0005515//protein binding	GO:0016568//chromatin modification;GO:0002759;GO:0006357//regulation of transcription from RNA polymerase II promoter;GO:0007444//imaginal disc development	gi|29841393|gb|AAP06425.1|/3.25519e-81/SJCHGC00614 protein

### KEGG pathway analysis

**Figure 3:**
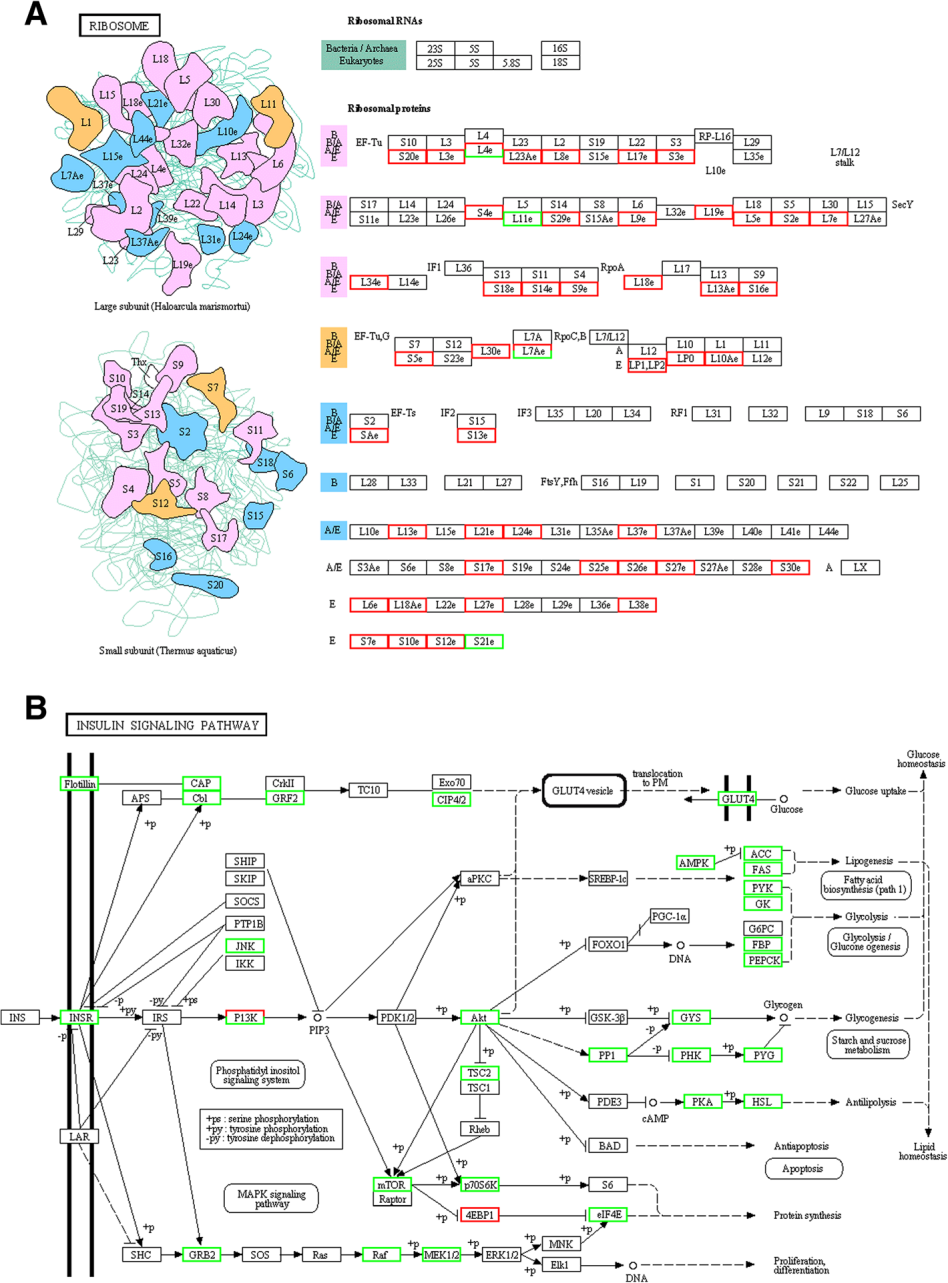
**KEGG pathway analysis. A.** Ribosome assembly. **B.** Insulin signalling pathway. Compared with the same genes in 23SSI, the up- and down-regulated genes are indicated with red and green colour in 23DSI, respectively.

KEGG pathway analysis was used to investigate the characteristics of the biochemical and metabolic processes involved with the differentially expressed genes. We found that these predicted target genes were involved in various metabolic processes, such as proteasome, porphyrin metabolism, ribosome, DNA replication, oxidative phosphorylation, pyrimidine metabolism, phagosome, folate biosynthesis, purine metabolism protein processing in the endoplasmic reticulum, aminoacyl-tRNA biosynthesis, protein digestion and absorption, basal transcription factors, glycerophospholipid metabolism, oocyte meiosis, neurotrophin signalling pathway, ubiquitin mediated proteolysis, RNA transport, peroxisome, basal transcription factors, apoptosis, glycerophospholipid metabolism, glycolysis/gluconeogenesis, citrate cycle, pentose phosphate pathway, fatty acid biosynthesis, and the insulin signalling pathway (Table 3). The predicted target genes of bantam hardly participated in the proteasome, porphyrin metabolism, ribosome, whereas more predicted target genes of miR-1-miR-71-miR-7-miR-7-5p were involved in these process. For instance, in ribosome assembly, 15 of 49 detected genes in this metabolic process were predicted as the target genes of miR-1-miR-71-miR-7-miR-7-5p, whereas only 1 of 49 genes was the predicted target gene of bantam (Figure 3A). However, none of the predicted target genes of miR-1-miR-71-miR-7-miR-7-5p are involved the citrate cycle, gastric acid secretion, glycolysis/gluconeogenesis, protein digestion and absorption, aminoacyl-tRNA biosynthesis, fatty acid biosynthesis, and the pentose phosphate pathway. Moreover, few of the predicted target genes of miR-1-miR-71-miR-7-miR-7-5p participated in the peroxisome, RNA degradation, mRNA surveillance pathway, axon guidance, basal transcription factors, apoptosis, glycerophospholipid metabolism, insulin signalling pathway, lysosome, regulation of actin cytoskeleton, and endocytosis. By contrast, more predicted target genes of bantam were involved in these processes. For example, among the 50 genes of the insulin signalling pathway, most of which were down-regulated in 23DSI. Out of the 50 genes, 33 were the predicted target genes of bantam (Figure 3B), whereas only 2 were predicted target genes of miR-1-miR-71-miR-7-miR-7-5p. In addition, in some processes such as DNA replication and oxidative phosphorylation, both participated in these processes on account of their similar amount of predicted target genes.

**Table 3:** Comparison of predicted target genes participating in regulating metabolic processes between miR-1- miR-71-miR-7-miR-7-5p and bantam

**Metabolic processes in KEGG**	**Detected genes participating in the process**	**Rate of predicted target genes of miR-1,miR-71,miR-7 and miR-7-5p**	**Rate of predicted target genes of bantam**
Proteasome	15	8(53%)	2 (13%)
Porphyrin metabolism	10	4 (40%)	3 (30%)
Ribosome	49	15 (31%)	1 (2%)
DNA replication	15	4 (27%)	3 (20%)
Oxidative phosphorylation	39	10 (26%)	11 (28%)
Pyrimidine metabolism	24	6 (25%)	11 (46%)
Phagosome	49	9 (18%)	26 (53%)
Folate biosynthesis	6	1 (17%)	3 (50%)
Purine metabolism	38	6 (16%)	20 (53%)
Protein processing in endoplasmic reticulum	68	9 (13%)	32 (47%)
Oocyte meiosis	32	4 (12%)	17 (53%)
Neurotrophin signaling pathway	25	3 (12%)	14 (56%)
Ubiquitin mediated proteolysis	54	6 (11%)	27 (50%)
RNA transport	62	6 (10%)	33 (53%)
Peroxisome	11	1 (9%)	4 (36%)
RNA degradation	35	3 (8%)	22 (63%)
mRNA surveillance pathway	35	3 (8%)	19 (54%)
Axon guidance	36	3 (8%)	25 (69%)
Basal transcription factors	15	1 (7%)	11 (73%)
Apoptosis	15	1 (7%)	9 (60%)
Glycerophospholipid metabolism	22	1 (5%)	12 (55%)
Insulin signaling pathway	50	2 (4%)	33 (66%)
Lysosome	61	2 (3%)	37 (61%)
Regulation of actin cytoskeleton	98	3 (3%)	57 (58%)
Endocytosis	68	2 (3%)	39 (57%)
Citrate cycle (TCA cycle)	13	0 (0%)	8 (62%)
Gastric acid secretion	19	0 (0%)	11 (58%)
Glycolysis/Gluconeogenesis	23	0 (0%)	11 (48%)
Protein digestion and absorption	21	0 (0%)	13 (62%)
Aminoacyl-tRNA biosynthesis	17	0 (0%)	10 (59%)
Fatty acid biosynthesis	3	0 (0%)	2 (67%)
pentose phosphate pathway	9	0 (0%)	6 (67%)

## Discussion

Pairing of *Schistosoma japonicum* initiates female development, leading to female sexual maturation, and the maintenance of this mature state. miRNAs are post-transcriptional regulators of growth and development in both plants and animals [18]. A comparison of the miRNA profiles of worms before and after pairing can provide insight into the mechanism by which pairing promotes female sexual maturation. During the development of *S. japonicum*, males and females just begin to pair about 18 d post-infection. At this stage, only some begin to pair. In this article, 18 d post-infection was also considered as the stage when pairing begins. Paired-females from double-sex infections begin to lay eggs about 24 d post-infection [30]. To rule out the influences of the egg, the 23-d-old female was considered herein as the earliest stage of sexual maturation after pairing. We compared the miRNA profiles among 18DSI, 18SSI, 23DSI, and 23SSI. We distinguished the effects of the pairing instead of the development from 18 d to 23 d post-infection. We found the level of high-abundance miRNAs such as miR-1, miR-1a, miR-1c, miR-71b-5p, and let-7 to be higher in 18DSI and 18SSI than in 23DSI and 23SSI. A similar miRNA profile between 18DSI and 18SSI was reasonable because both of them stayed nearly at the same stage at 18 d post-infection. Although some paired, they likely paired for a short while, indicating that males did not play a distinct role in paired-females.

At 5 d post-pairing, 23DSI was significantly longer and thicker than 23SSI. Moreover, 23 DSI had a dark brown colour because of the accumulation of larger amounts of schistosome hemozoin in their guts. By contrast, 23SSI was smaller and hardly accumulated schistosoma hemozoin in their guts [5]. Interestingly, these distinct morphological changes appeared to bring about limited changes in the miRNA profiles. However, these limited differentially depressed miRNAs were possibly associated with some significant developmental events regarding sexual maturation. We found that the expression profile of miRNA in 23DSI is a little similar to that of 23SSI. In particular, nearly all high-abundance miRNAs, such as miR-1c, miR-1a, miR-10-5p, miR-71b-5p, and let-7, were down-regulated in both, compared with 18DSI or 18SSI. Only several high-abundance miRNAs differentially expressed between 23DSI and 23 SSI, such as bantam, miR-1, miR-71, miR-7, and miR-7-5p. These results suggested that high-abundance miRNAs such as miR-1c, miR-1a, miR-10-5p, miR-71b-5p, and let-7 were closely related to the development of 18 d-old females before pairing, whereas during the development from 18 d to 23 d, all of these high-abundance miRNAs were down-regulated not only in 23 DSI, but also in 23SSI. This trend indicated that the up- or down-regulation of all of these high-abundance miRNAs were not related to the pairing but only to female development. However, the up-regulation of bantam in 23DSI was associated not only with pairing but also likely played an essential role in female sexual maturation.

To investigate the role of these differentially expressed miRNAs in 23 DSI and 23SSI, we analysed their predicted target genes and relevant functions. Moreover, KEGG pathway analysis was used to investigate the various metabolic processes involved in these predicted target genes. We found that the target genes of bantam were involved in widely different metabolic processes, such as in peroxisome, RNA degradation, mRNA surveillance pathway, axon guidance, basal transcription factors, apoptosis, glycerophospholipid metabolism, insulin signalling pathway, lysosome, regulation of actin cytoskeleton, citrate cycle, gastric acid secretion, glycolysis/gluconeogenesis, protein digestion and absorption, aminoacyl-tRNA biosynthesis, and fatty acid biosynthesis. In 23DSI, the up-regulation of bantam likely inhibited a large number of genes or pathways involved within a wide range of biological functions, including glycometabolism, lipid metabolism, nucleic acid metabolism, protein digestion and utilisation, and other biological processes. This result was consistent with findings on gene expression analysis [5].

In unpaired females (23SSI), bantam was notably not up-regulated, whereas miR-1, miR-71, miR-7, and miR-7-5p were significantly up-regulated. By contrast, in paired females (23DSI), the above mentioned miRNAs were not up-regulated, suggesting that the functions of the target genes of miR-1-miR-71-miR-7-miR-7-5p were required in paired females. We found that the target genes of miR-1-miR-71-miR-7-miR-7-5p, such as ribosomal protein genes (CAX72037.1, CAX71939.1, CAX78482.1, CAX77178.1, AAP06483.1, CAX77387.1, CAX72859.1, CAX70956.1, CAX71543.1, CAX83047.1, CAX70121.1) (Additional file 6: Table S4), thioredoxin peroxidase (CAX75860.1), tubulin (XP_002580033.1, CAX75788.1, CAX75500.1, CAX71989.1, CAX76110.1), ATP synthase- H + transporting (CAX75390.1, CAX76063.1), and cytochrome c oxidase (CAX74747.1, CAX76589.1), among others, were significantly up-regulated. In particular, various ribosomal protein genes were regulated by miR-1-miR-71-miR-7-miR-7-5p. These results suggested that miR-1, miR-71, miR-7, and miR-7-5p played an essential role in regulating ribosomal assembly. Hence, enhancing the ribosomal assembly leads to the enhancement of protein expression, suggesting that, although more genes and biological processes were inhibited in paired females, enhanced ribosomal assembly likely maintains the supply of a large amount of special proteins for sexual maturation and egg production. In 23 DSI, the high level of bantam and low levels of miR-1, miR-71, miR-7, and miR-7-5p possibly regulated and organised a specific gene expression profile for sexual maturation and egg production by inhibiting and strengthening specific gene expression and metabolic processes. Our previous research has shown that genes coding for proteins such as phosphoglycerate mutase, superoxide dismutase, egg antigen, ribosomal protein, ferritin-1 heavy chain, and eukaryotic translation initiation factor 2 were significantly up-regulated in 23DSI. These genes function in glycolysis, antioxidant defense, protein biosynthesis, egg formation, iron transport and utilisation, and translational regulation (data not shown). The above mentioned proteins appeared to be more necessary for egg production. Other previous studies have compared differential gene expression between females and males, and similar results have also been reported. For example, egg antigen [5,40,41], ferritin-1 [5,40,42,43], ribosomal proteins [5,40,44], ATPase [44,45], cathepsin [45], extracellular superoxide dismutase [5,43,44], cytochrome C oxidase [5], tyrosinase [43], mucin-like protein [46], fs800 [47], and adenylosuccinate lyase [5], among others, are often detected in females. These up-regulated genes in paired females are considered to play roles in female sexual maturation and egg production. Only some of them belong to the predicted target genes of differentially expressed miRNAs, suggesting that miRNAs of 23DSI do not regulate all genes relate to sexual maturation and egg production.

Previous studies have reported sexual differences in the gene expression of schistosomes [5,6,40,43,48-50]. However, how pairing induces changes in the gene expression of females is unclear. Although miRNAs do not regulate all genes in organisms, evidence provided by miRNA analyses in the present study indicated that pairing likely limited the expression of non-essential genes through increasing the expression of bantam and specific genes by maintaining miR-1, miR-71, miR-7, and miR-7-5p at relatively low levels. Interestingly, miR-71 was located on the female W chromosome, suggesting mir-71 be involved in female sex-specific functions [51]. Thus, the change of the abundance of miR-71 in worms maybe plays a key role in female development. In addition, mass MALDI-TOF-TOF MS analyses have also shown that fatty acid-binding protein and phosphoglycerate mutase can be detected in paired 19-d-old females, and ribosomal protein LP1 [52] and ribosomal protein L30 [53] can be detected in paired 42-d-old females. These results further revealed that these up-regulated genes in 23 DSI detected by DGE and predicted as target genes of differentially expressed miRNAs can be translated to proteins, such as ribosomal proteins. Thus, the regulation of ribosome assembly by miRNA likely played a significant role in sexual maturation and egg production in paired females.

After pairing, the up-regulation of bantam was potentially capable of inhibiting a wide range of genes or biological processes. Furthermore, the low abundance of miR-1, miR-71, miR-7, and miR-7-5p in 23DSI compared with 23SSI was likely capable of promoting specific gene expression. The function of their target genes and relevant biological processes were consistent with evidence observed from gene expression experiments. These results suggested that pairing facilitated female sexual maturation and egg production by regulating miRNA profiles, and thus, gene expression.

## Conclusions

The differentially expressed miRNAs between 23SSI and 23DSI and their different functions indicated that more genes or metabolic pathways in paired mature females were inhibited than those in unpaired immature females. The results suggested that specific miRNAs regulated gene expression to lead to female sexual maturation after pairing.

## Competing interests

The authors declare that they have no competing interests.

## Authors’ contributions

Collected material, bioinformatic analysis and quantitative RT-PCR analysis: JS, SW, CL, YR, JW. Analyzed the data and performed statistical evaluation: JS, SW. Wrote the paper: JS. All authors read and approved the final version of the manuscript.

## Supplementary Material

Additional file 1 Analysis of differentially expressed miRNAs. Click here for file

Additional file 2 Analysis of differentially expressed genes. Click here for file

Additional file 3: Table S1 Predicted target genes of miR-1-miR-71-miR-7-miR-7-5p in *Schistosoma japonicum.* Click here for file

Additional file 4: Table S2 Predicted target genes of bantam in *Schistosoma japonicum*. Click here for file

Additional file 5: Table S3 Predicted target genes of bantam in down-regulated genes in 23DSI. Click here for file

Additional file 6: Table S4 Predicted target genes of miR-1-miR-71-miR-7-miR-7-5p in up-regulated genes in 23DSI. Click here for file
