# Supplementary material for: Novel expression profiles of microRNAs suggest that specific miRNAs regulate gene expression for the sexual maturation of female Schistosoma japonicum after pairing
Source: Parasit Vectors. 2014 Apr 10;7:177. doi: 10.1186/1756-3305-7-177 (PMC4021575; doi:10.1186/1756-3305-7-177)
Supplement: Additional file 1 — Analysis of differentially expressed miRNAs. [file 1756-3305-7-177-S1.docx]

**Complementary material 1**

- Basic principle

    Small RNAs (sRNA) from HiSeq deep sequencing cover almost every kind of RNA, including miRNA, siRNA, piRNA, rRNA, tRNA, snRNA, snoRNA，repeat associated sRNA and degraded tags of exon or intron. By comparing our sequences with those in databases and picking out the overlap on genome location between our data and the databases, sRNAs can be annotated into different categories. Those which can not be annotated will be used to predict novel miRNA by the self-developed software Mireap.

- Work Flow

1. Experiment process

Small RNA is an special kind of molecules in organisms which induces the gene silence and plays an important role in the regulation of cell growth, gene transcription and translation.

The small RNA digitalization analysis based on HiSeq high-throughput sequencing takes the SBS-sequencing by synthesis, which can decrease the loss of nucleotides caused by the secondary structure. It is also strong for its small requirement of sample quantity, high through-put, high accuracy with simply operated automatic platform. Such analysis can obtain millions of small RNA sequence tags in one shot, identify small RNA of certain species in certain condition comprehensively, predict novel miRNA and construct the small RNA differential expression profile between samples, which could be used as a powerful tool on small RNA function research. The experiment process^1^ of small RNA sequencing is shown in Figure1 and Figure 2:

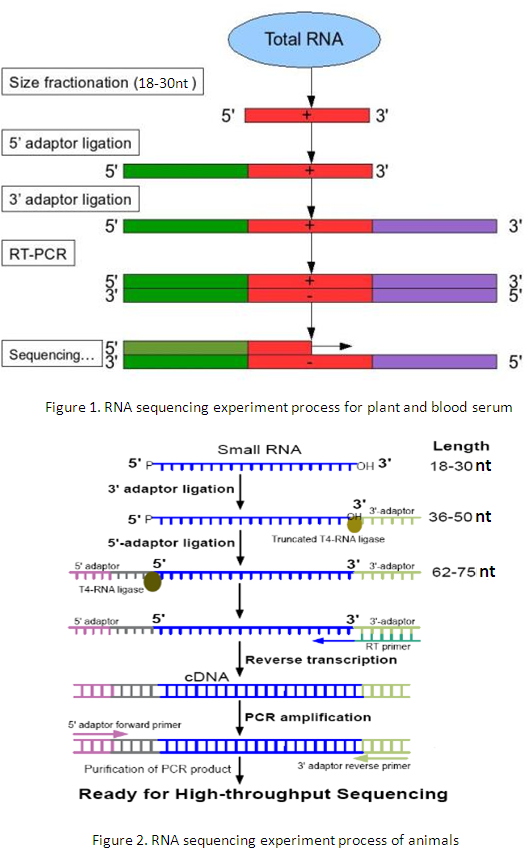


1. Data analysis process

The 50nt sequence tags from HiSeq sequencing will go through the data cleaning first, which includes getting rid of the low quality tags and several kinds of contaminants from the 50nt tags. Length distribution of clean tags are then summarized. Afterwards, the standard bioinformatics analysis will annotate the clean tags into different categories and take those which can not be annotated to any category to predict the novel miRNA and base edit of potential known miRNA. The whole process is shown as below:

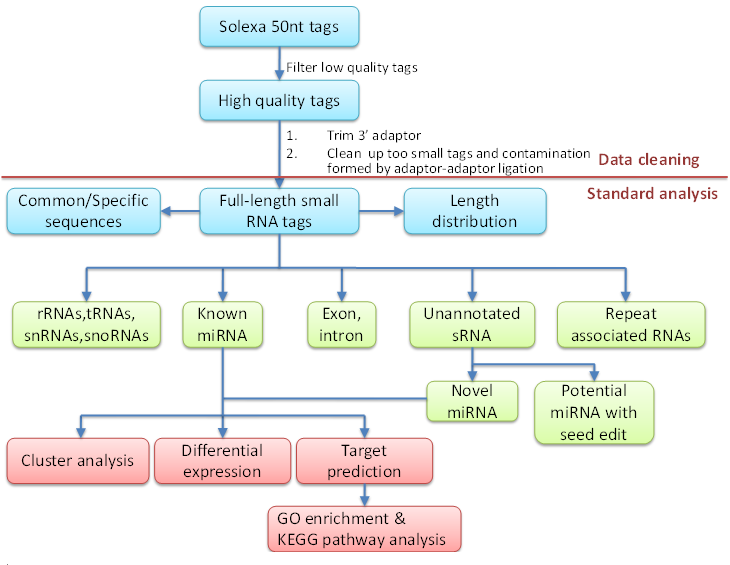


- Instructions on result

1. Raw Data

The very basic figure from sequencing is converted into sequence data by the base calling step. Such sequence data called raw data or raw reads is stored in the *.fq file under /sample_name/result_primary in a fastq format. In a fastq format file, one sequence tag (called one "read") is represented by four lines:

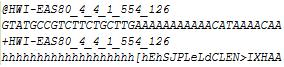

The first and third lines are names of this read, generated by sequencing machine (usually the names will be coded by random characters but not the names). The second line is the sequence. The fourth line represents the sequencing quality of this read. Each character in this line shows the sequencing quality of the base on the same position in the second line. The actual quality is the corresponding ASCII value of the letter minus 64. For example, the ASCII value of letter "i" is 104. So the sequencing quality of bases with letter i in the fourth line is 105-64=41. The quality of HiSeq sequencing ranges from 0 to 41. This quality will be used in the criteria for filtering out low quality reads. Table 3 shows some common situations of sequencing error rate and sequencing quality correspondence. The relationship between sequencing error rate (E) and sequencing quality (sQ) is shown in the below formula:

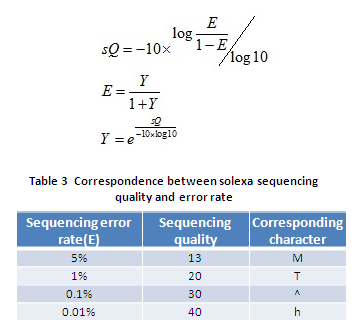


1. Data cleaning and length distribution

We will get rid of some contaminant reads from the fq file and get the final clean reads. And then summarize the length distribution of these clean reads. Normally, length of small RNA is between 18nt and 30nt. The length distribution analysis is helpful to see the compositions of small RNA sample. For example, miRNA is normally 21nt or 22nt, siRNA is 24nt, and piRNA is 30nt.

The data is processed by the following steps:
1) Getting rid of low quality reads (The criteria for this is listed in the explanation of meaning of each row in the result table)
2) Getting rid of reads with 5' primer contaminants
3) Getting rid of reads without 3' primer
4) Getting rid of reads without the insert tag
5) Getting rid of reads with poly A
6) Getting rid of reads shorter than 18nt
7) Summarize the length distribution of the clean reads

**Program and Parameters:**
We use software developed by BGI to deal with the data from HiSeq sequencing.

Result table and figure: Click the figure in the result page to see the high definition figure.

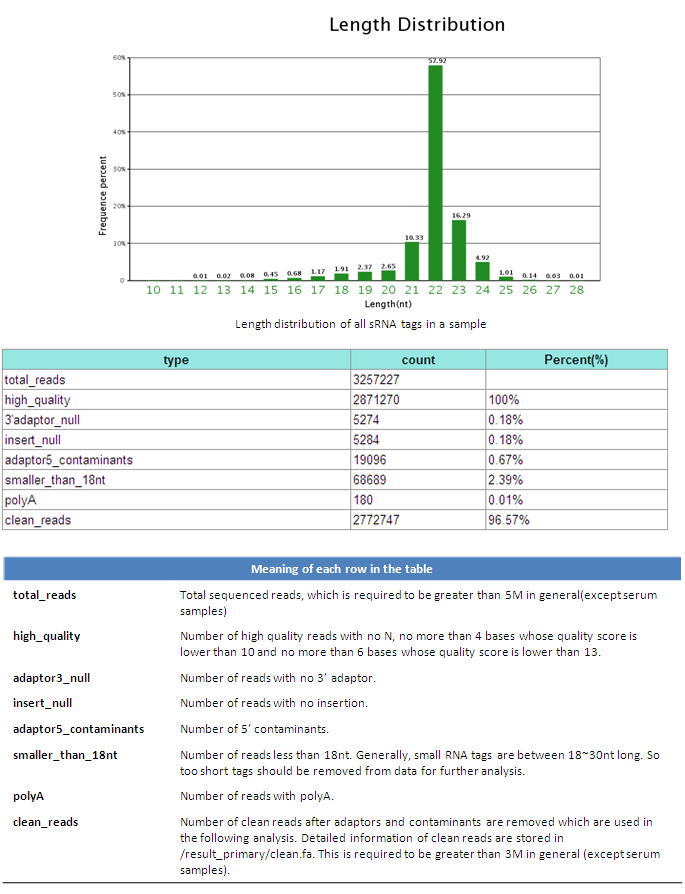


1. Mapping to genome

Map the small RNA tags to genome by SOAP to analyze their expression and distribution on the genome.

**Program and Parameters:**
soap -v 0 -r 2 -M 0 -a clean.fa -D ref_genome.fa.index -o match_genome.soap

Result table and figure: Click the figure in the result page to see the high definition figure.

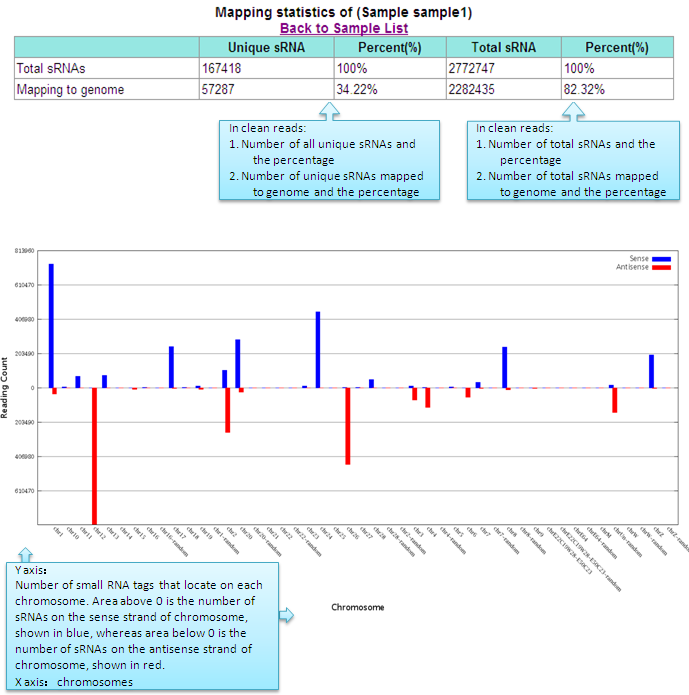


1. Known miRNA expression profile

(1)If there is miRNA information of the species in miRBase81
Align small RNA tags to the miRNA precursor/mature miRNA of corresponding species in miRBase18. Show detailed information of alignment, including structure of known miRNA precursor, length and count of tags from the sample, etc. Click the miRNA id in the left table to see detailed information of that miRNA.

**Program and Parameters:**
blastall -p blastn -F F -e 0.01
Result table and figure: Click the figure in the result page to see the high definition figure.

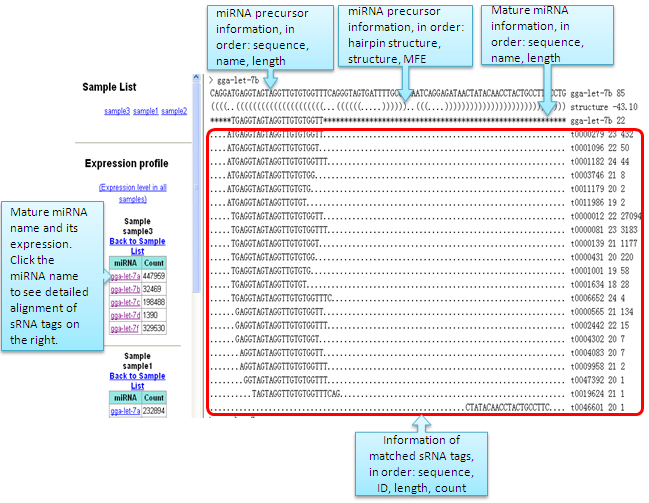


(2)If there is no miRNA information of the species in miRBase18
Align small RNA tags to the miRNA precursor/mature miRNA of all plants/animals in miRBase18. Show the sequence and count of miRNA families(no specific species) which can be found in the samples。

**Program and Parameters:**
Software developed by BGI- tag2miRNA
Result table and figure: Click the figure in the result page to see the high definition figure.

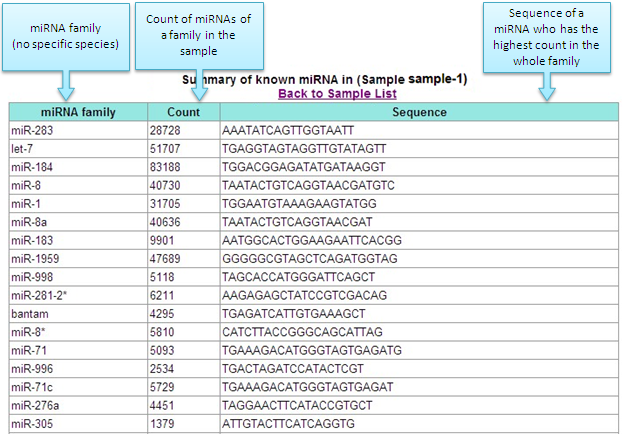


1. Alignment to Genbank

Annotate the small RNA tags with rRNA, scRNA, snoRNA, snRNA and tRNA from Genbank and get rid of matched tags from unannotated tags.

**Program and Parameters:**
blastall -p blastn -F F -e 0.01

Result table and figure: Click the figure in the result page to see the high definition figure.

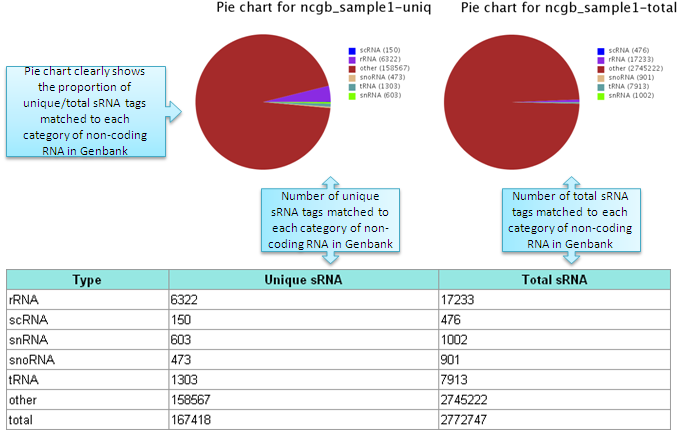


1. Alignment to Rfam

Annotate the small RNA tags with sequences from Rfam and get rid of matched tags from unannotated tags.

**Program and Parameters:**
blastall -p blastn -F F -e 0.01

1. Exon and intron alignment

Align small RNA tags to exons and introns of mRNA to find the degraded fragments of mRNA in the small RNA tags.

**Program and Parameters:**
Software developed by BGI - overlap

1. Small RNA annotation

Summarize all alignments and annotation before. In the alignment and annotation before, some small RNA tags may be mapped to more than one categories. To make every unique small RNA mapped to only one annotation, we follow the following priority rule:rRNAetc (in which Genbank > Rfam) > known miRNA > repeat > exon > intron^3^. The total rRNA proportion is a mark for sample quality check. Usually it should be less than 60% in plant samples and 40% in animal samples as high quality.

**Program and Parameters:**
Software developed by BGI - tag2annotation
Result table and figure: Click the figure in the result page to see the high definition figure.

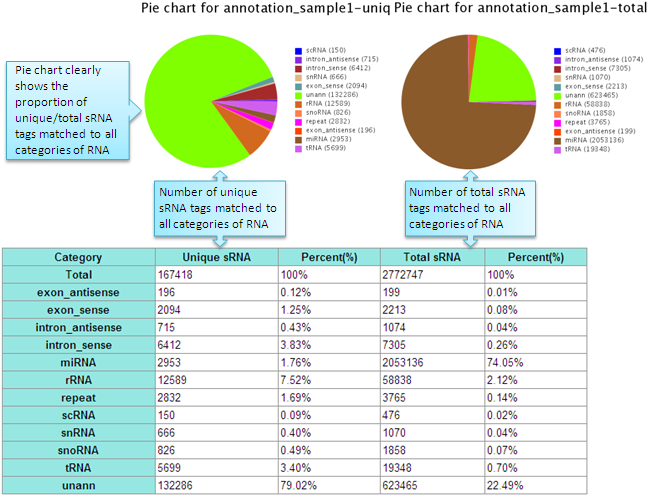


1. Differential expression of known miRNA

Compare the known miRNA expression between two samples to find out the differentially expressed miRNA. Show the expression of miRNA in two samples by plotting Log2-ratio figure and Scatter Plot. The procedures are shown as below:
(1)Normalize the expression of miRNA in two samples (control and treatment) to get the expression of transcript per million (TPM).
    Normalization formula: Normalized expression = Actual miRNA count/Total count of clean reads*1000000 ,
(2)Calculate fold-change and P-value from the normalized expression. Then generate the log2ratio plot and scatter plot.
    Fold-change formula: Fold_change=log2(treatment/control)
    P-value formula:

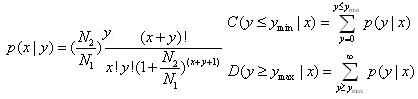


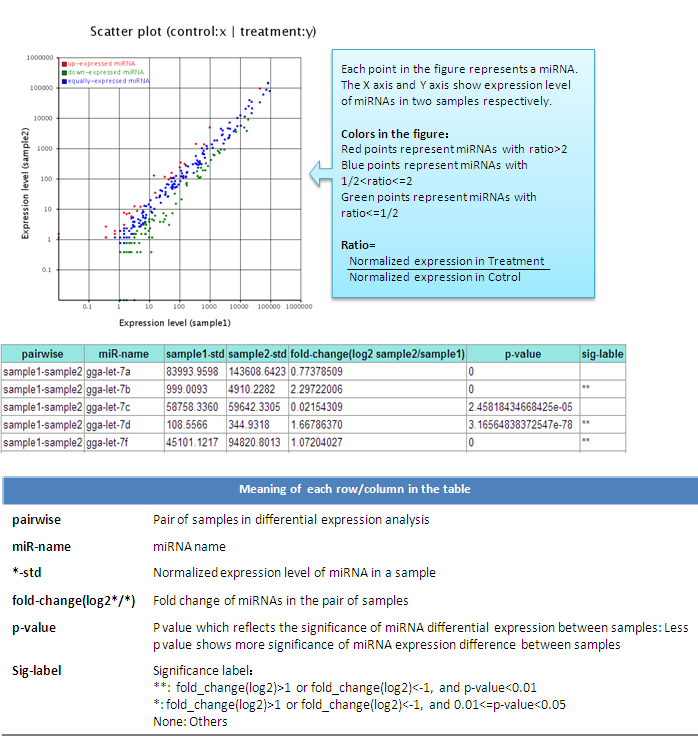


1. Target prediction for known miRNA
   Predict the target gene of novel miRNA from Mireap and show the number of miRNA (only miRNA with predicted target genes will be included here) and number of corresponding target genes in the result.

The rules used for target prediction are based on those suggested by Allen et al.^5^ and Schwab et al.^6^:
(1)No more than four mismatches between sRNA & target (G-U bases count as 0.5 mismatches)
(2)No more than two adjacent mismatches in the miRNA/target duplex
(3)No adjacent mismatches in in positions 2-12 of the miRNA/target duplex (5' of miRNA)
(4)No mismatches in positions 10-11 of miRNA/target duplex
(5)No more than 2.5 mismatches in positions 1-12 of the of the miRNA/target duplex (5' of miRNA)
(6)Minimum free energy (MFE) of the miRNA/target duplex should be >= 75% of the MFE of the miRNA bound to it's perfect com plement

1. KEGG pathway analysis

KEGG pathway analysis is also used for the target gene candidates. In organisms, genes usually interact with each other to play different roles in certain biological function. The analysis based on pathways could facilitate the understanding of biological functions of genes. KEGG is the major public pathway-related database[7]. KEGG pathway analysis identifies significantly enriched metabolic pathways or signal transduction pathways in target gene candidates comparing with the whole reference gene background. The calculating formula is the same as that in GO analysis. Here N is the number of all genes with KEGG annotation, n is the number of target gene candidates in N, M is the number of all genes annotated to a certain pathway, and m is the number of target gene candidates in M. Genes with FDR≤0.05 are considered as significantly enriched in target gene candidates. The KEGG analysis could reveal the main pathways which the target gene candidates are involved in.

- Reference

1. Hafner, M., P. Landgraf, et al. (2008). "Identification of microRNAs and other small regulatory RNAs using cDNA library sequencing." Methods 44(1): 3-12.
2. Ruby, J. G., C. Jan, et al. (2006). "Large-scale sequencing reveals 21U-RNAs and additional microRNAs and endogenous siRNAs in C. elegans." Cell 127(6): 1193-207.
3. Calabrese, J. M., A. C. Seila, et al. (2007). "RNA sequence analysis defines Dicer's role in mouse embryonic stem cells". Proc Natl Acad Sci USA 104(46): 18097-102.
4. Zhang, Y., X. Zhou, et al. (2009). "Insect-Specific microRNA Involved in the Development of the Silkworm Bombyx mori." PLoS One 4(3): e4677.
5. Allen, E., Z. Xie, et al. (2005). "microRNA-directed phasing during trans-acting siRNA biogenesis in plants." Cell 121(2): 207-21.
6. Schwab, R., J. F. Palatnik, et al. (2005). "Specific effects of microRNAs on the plant transcriptome." Dev Cell 8(4): 517-27.
7. Kanehisa, M., M. Araki, et al. (2008). "KEGG for linking genomes to life and the environment." Nucleic Acids Res. 36 (Database issue): D480-4.
