# Supplementary material for: Novel expression profiles of microRNAs suggest that specific miRNAs regulate gene expression for the sexual maturation of female Schistosoma japonicum after pairing
Source: Parasit Vectors. 2014 Apr 10;7:177. doi: 10.1186/1756-3305-7-177 (PMC4021575; doi:10.1186/1756-3305-7-177)
Supplement: Additional file 2 — Analysis of differentially expressed genes. [file 1756-3305-7-177-S2.docx]

**Complementary material**

The experimental process includes sample preparation and sequencing. The main reagents and supplies are Illumina Gene Expression Sample Prep Kit and Solexa Sequencing Chip (flowcell), and the main instruments are Illumina Cluster Station and Illumina HiSeq™ 2000 System.

Specified Experimental Process: Extract 6 µg total RNA, use Oligo(dT) magnetic beads adsorption to purify mRNA, and then use Oligo(dT) as primer to synthesize the first and second-strand cDNA. The 5' ends of tags can be generated by two types of Endonuclease: *Nla*III or *Dpn*II (see table 1 for recognition sites). Usually, the bead-bound cDNA is subsequently digested with restriction enzyme *Nla*III, which recognizes and cuts off the CATG sites. The fragments apart from the 3' cDNA fragments connected to Oligo(dT) beads are washed away and the Illumina adaptor 1 is ligated to the sticky 5' end of the digested bead-bound cDNA fragments. The junction of Illumina adaptor 1 and CATG site is the recognition site of *Mme*I, which is a type of Endonuclease with separated recognition sites and digestion sites. It cuts at 17bp downstream of the CATG site, producing tags with adaptor 1. After removing 3' fragments with magnetic beads precipitation, Illumina adaptor 2 is ligated to the 3' ends of tags, acquiring tags with different adaptors of both ends to form a tag library. After 15 cycles of linear PCR amplification, 105bp fragments are purified by 6% TBE PAGE Gel electrophoresis. After denaturation, the single-chain molecules are fixed onto the Illumina Sequencing Chip (flowcell). Each molecule grows into a single-molecule cluster sequencing template through Situ amplification. Then add in four types of nucleotides which are labeled by four colors, and perform sequencing with the method of sequencing by synthesis (SBS). Each tunnel will generate millions of raw reads with sequencing length of 49bp.

**Pipeline of bioinformatics analysis**


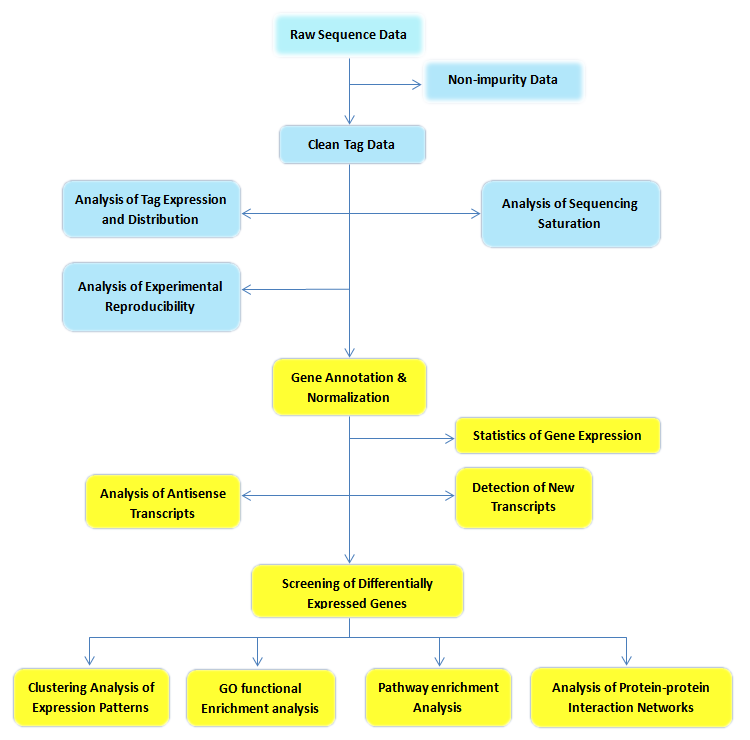

Figure 1. Procedure of bioinformatics analysis for digital gene expression profiling

**Raw data**[**back to Pipeline of bioinformatics analysis**](file:///D:\单23分析\analysis2-predicted%20and%20sj%20mansoni\upload\index_en.html#it)

Sequencing-received raw image data is transformed by base calling into sequence data, which is called raw data or raw reads, is stored in FASTQ format.

**Filter dirty tags**[**back to Pipeline of bioinformatics analysis**](file:///D:\单23分析\analysis2-predicted%20and%20sj%20mansoni\upload\index_en.html#it)

Raw sequences have 3' adaptor fragments as well as a few low-quality sequences and several types of impurities. Raw sequences are transformed into Clean Tags after certain steps of data-processing.

**Data-processing steps**

1. 3' adaptor sequence removal: since Tags are only 21nt long while the sequencing reads are 49nt long, raw sequences are with 3' adaptor sequences
2. Empty reads removal (reads with only 3' adaptor sequences but no Tags)
3. Low quality Tags removal (Tags with unknown sequences 'N')
4. Removal of Tags which are too long or too short, leaving Tags of 21nt long
5. Removal of Tags with a copy number of 1 (probably be sequencing error)
6. Generate Clean Tags

**Clean Tag**[**back to Pipeline of bioinformatics analysis**](file:///D:\单23分析\analysis2-predicted%20and%20sj%20mansoni\upload\index_en.html#it)

Tags after [filtering dirty tags](file:///D:\单23分析\analysis2-predicted%20and%20sj%20mansoni\upload\index_en.html#clean) from raw data.

**Saturation analysis of sequencing**[**back to Pipeline of bioinformatics analysis**](file:///D:\单23分析\analysis2-predicted%20and%20sj%20mansoni\upload\index_en.html#it)

The saturation analysis can be performed to check whether the number of detected genes keep increasing when sequencing amount (total tag number) increases. Example in figure 2 shows a trend of saturation. When sequencing amount reaches 2M or higher, the number of detected genes almost ceases to increase.


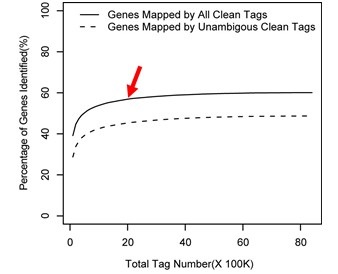

Figure 2. Saturation evaluation of different expression

**Experimental repeatability analysis**[**back to Pipeline of bioinformatics analysis**](file:///D:\单23分析\analysis2-predicted%20and%20sj%20mansoni\upload\index_en.html#it)

Correlation analysis of two parallel experiments provides the evaluation of the reliability of experimental results as well as operational stability. Figure 6 is an example that shows the correlation of two parallel experiments. The closer the value of correlation gets to 1, the better the reproducibility between two parallel experiments.


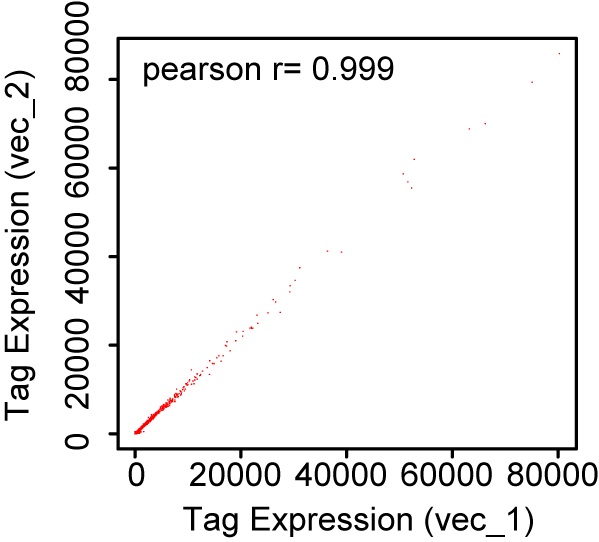

Figure 3. Reproducibility analysis of two independent experiments

**Gene expression annotation**[**back to Pipeline of bioinformatics analysis**](file:///D:\单23分析\analysis2-predicted%20and%20sj%20mansoni\upload\index_en.html#it)

A virtual libraries containing all the possible CATG+17 bases length sequences of the reference gene sequences (if there are no reference gene sequences for the species, sequences from closely related species can be used for reference). All clean tags were mapped to the reference sequences and only 1bp mismatch is considered. Clean tags mapped to reference sequences from multiple genes were filtered. Remainder clean tags were designed as unambiguous clean tags. The number of unambiguous clean tags for each gene was calculated and then normalized to TPM (number of transcripts per million clean tags). (['t Hoen, Ariyurek, *et al*. 2008](file:///D:\单23分析\analysis2-predicted%20and%20sj%20mansoni\upload\index_en.html#cite1); [Morrissy, Morin, *et al*. 2009](file:///D:\单23分析\analysis2-predicted%20and%20sj%20mansoni\upload\index_en.html#cite2)). Below is an example (Table 1).

Table 1. Raw gene expression and standardized gene expression (TPM)

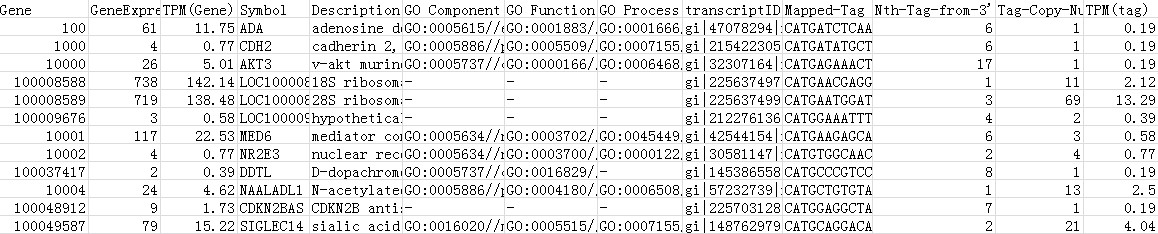


| **Each column stands for:** | |
| --- | --- |
| Gene | Gene ID |
| GeneExpression | Raw clean tag number |
| TPM(Gene) | Sum of the normalized clean tag number |
| Symbol | Gene Symbol |
| Description | Brief gene description |
| Pathway | KEGG Pathway |
| GO Component | Ontology Information of Cellular Components of Gene-corresponding GO terms |
| GO Function | Ontology Information of Molecular Functions of Gene-corresponding GO terms |
| GO Process | Ontology Information of Biological Processes of Gene-corresponding GO terms |
| blast nr | result of blast against nr |
| transcriptID | Gene Names in Transcripts Sequences |
| Mapped-Tag | Tag sequences that uniquely mapped to genes |
| Nth-Tag-from-3'-end-of-Gene | The Nth "CATG" site from 3' end of gene |
| Tag-Copy-Number | Raw tag copy number |
| TPM (tag) | Normalized tag copy number |

Notice: TPM (Transcripts Per Million clean tags) is a standardized indicator, pointing out number of transcript copies in every 1 million clean tags. The four columns of Symbol, Description, blast nr and transcriptID do not appear in every result; part of these four columns will be integrated into reference gene information. Please refer to your specific result for final results.

**Statistics of Clean Tags**[**back to Pipeline of bioinformatics analysis**](file:///D:\单23分析\analysis2-predicted%20and%20sj%20mansoni\upload\index_en.html#it)

Table 2. Raw expression of the Clean Tag and standardized tag expression

| **Tag** | **CopyNumber** | **TPM(Transcript Per Million)** |
| --- | --- | --- |
| CATGTATCCTGAAAATATGCA | 2 | 0.43 |
| CATGTCTCAAGGGGTTAGCTT | 2 | 0.43 |
| CATGAGAATCGGTTGGACTTA | 2 | 0.43 |
| CATGCCACTGCACTCCATCTT | 2 | 0.43 |
| CATGGGAGGGAGAGCAAGCAC | 2 | 0.43 |
| CATGGCATTTCTCAATAAAAA | 2 | 0.43 |
| CATGAGAGATATTGGTCTATA | 2 | 0.43 |
| CATGCTTATTATGGTTTTTGT | 2 | 0.43 |
| CATGTTTTGCTCTGCAAAAGA | 2 | 0.43 |
| CATGCCATTTTTAAGGGGTTC | 2 | 0.43 |

Note: TPM (Transcripts Per Million clean tags) is a standardized indicator, pointing out number of transcript copies in every 1 million clean tags.

**Screening of differentially expressed genes**[**back to Pipeline of bioinformatics analysis**](file:///D:\单23分析\analysis2-predicted%20and%20sj%20mansoni\upload\index_en.html#it)

Referring to "The significance of digital gene expression profiles" ([Audic, Claverie. 1997](file:///D:\单23分析\analysis2-predicted%20and%20sj%20mansoni\upload\index_en.html#cite3)).

Denote the number of unambiguous clean tag from gene A as x, as every gene's expression occupies only a small part of the library, the p(x) is in the Poisson distribution.


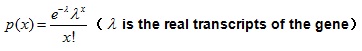


The total clean tag number of the sample 1 is N1, and total clean tag number of sample 2 is N2; gene A holds x tags in sample1 and y tags in sample2. The probability of gene A expressed equally between two samples can be calculated with:


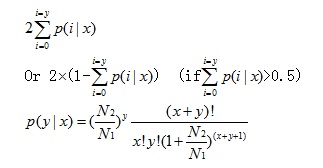


P Value corresponds to differential gene expression test. FDR (False Discovery Rate) is a method to determine the threshold of P Value in multiple test and analysis through manipulating the FDR value. Assume that we have picked out R differentially expressed genes in which S genes are really show differential expression and the other V genes are false positive. If we decide that the error ratio "Q=V/R" must stay below a cutoff (e.g. 1%), we should preset the FDR to a number no larger than 0.01. Refer to [Benjamini, Yekutieli. 2001](file:///D:\单23分析\analysis2-predicted%20and%20sj%20mansoni\upload\index_en.html#cite4) for details. We use "FDR≤0.001 and the absolute value of log2Ratio≥1" as the threshold to judge the significance of gene expression difference. More stringent criteria with smaller FDR and bigger fold-change value can be used to identify DEGs.

Table 3. List of Differentially Expressed Genes (DEGs)

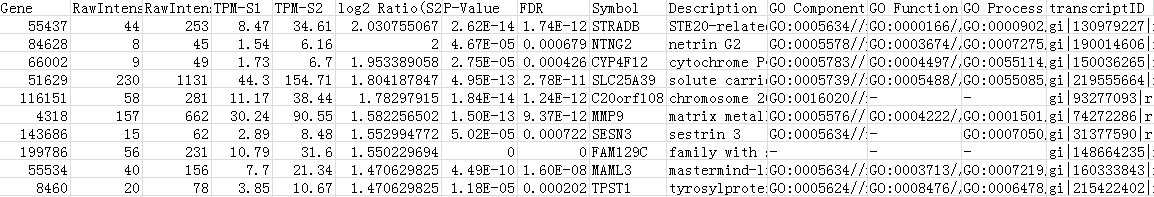


| **Each column stands for:** | |
| --- | --- |
| Gene | Gene ID |
| RawIntensity-S1 | Raw expression level of genes in sample "S1" |
| RawIntensity-S2 | Raw expression level of genes in sample "S2" |
| TPM-S1 | Normalized expression level of genes in sample "S1" |
| TPM-S2 | Normalized expression level of genes in sample "S2" |
| log2 Ratio(S2/S1) | log2(multiples of differentially expressed) |
| P-Value | P-value from difference test |
| FDR | FDR value |
| Symbol | Gene Symbol |
| Description | Brief gene description |
| Pathway | KEGG Pathway |
| GO Component | Ontology Information of Cellular Components of Gene-corresponding GO terms |
| GO Function | Ontology Information of Molecular Functions of Gene-corresponding GO terms |
| GO Process | Ontology Information of Biological Processes of Gene-corresponding GO terms |
| blast nr | result of blast against nr |
| transcriptID | Gene Names in Transcripts Sequences |

Notice: The four columns of Symbol, Description, blast nr and transcriptID do not appear in every result; part of these four columns will be integrated into reference gene information. Please refer to your specific result for final results.

**Gene Ontology Functional Enrichment Analysis for DEGs**[**back to Pipeline of bioinformatics analysis**](file:///D:\单23分析\analysis2-predicted%20and%20sj%20mansoni\upload\index_en.html#it)

Gene Ontology (GO) is an international standardized gene functional classification system which offers a dynamic-updated controlled vocabulary and a strictly defined concept to comprehensively describe properties of genes and their products in any organism. GO has three ontologies: molecular function, cellular component and biological process. The basic unit of GO is GO-term. Every GO-term belongs to a type of ontology.

In gene expression profiling analysis, GO enrichment analysis of functional significance applies hypergeometric test to map all differentially expressed genes to terms in GO database, looking for significantly enriched GO terms in DEGs comparing to the genome background. The calculating formula is:


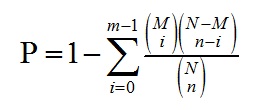


Where N is the number of all genes with GO annotation; n is the number of DEGs in N; M is the number of all genes that are annotated to the certain GO terms; m is the number of DEGs in M.

Our GO functional enrichment analysis also integrates the clustering analysis of expression patterns. Thus, researchers can easily get the expression patterns of DEGs annotated to the given GO-term. For example, Immune system process is the most significantly enriched GO term in DEGs. Figure 10 shows the expression patterns of the involved DEGs.

Table 4. Significantly enriched GO terms in DEGs

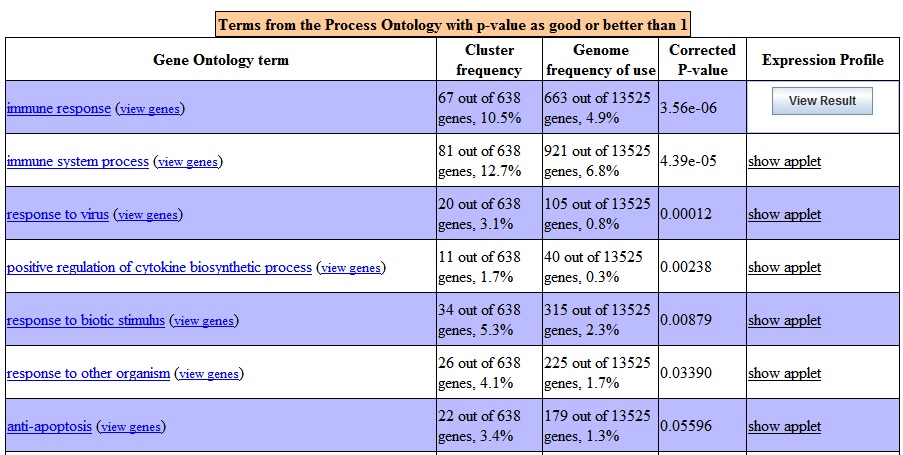


**Pathway Enrichment Analysis for DEGs**[**back to Pipeline of bioinformatics analysis**](file:///D:\单23分析\analysis2-predicted%20and%20sj%20mansoni\upload\index_en.html#it)

Different genes usually cooperate with each other to exercise their biological functions. Pathway-based analysis helps to further understand genes biological functions. KEGG is the major public pathway-related database. Pathway enrichment analysis identifies significantly enriched metabolic pathways or signal transduction pathways in DEGs comparing with the whole genome background. The calculating formula is the same as that in GO analysis. Here N is the number of all genes that with KEGG annotation, n is the number of DEGs in N, M is the number of all genes annotated to specific pathways, and m is number of DEGs in M. The results are as Table 5:

Table 5. List of Pathway Enrichment Analysis

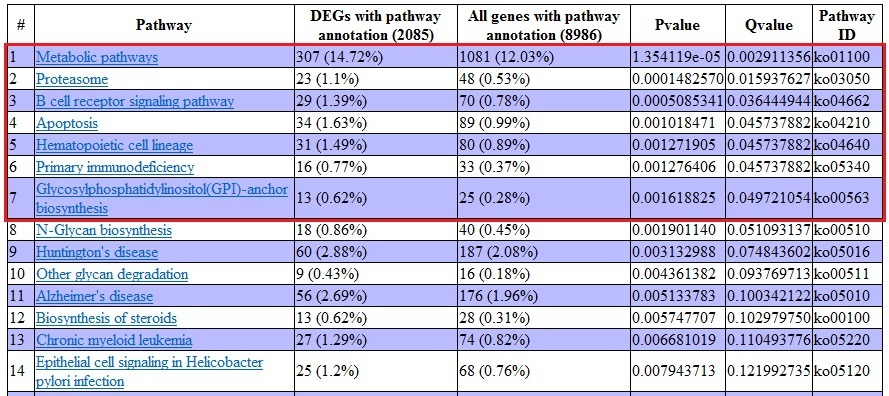


| **Each column stands for:** | |
| --- | --- |
| # | No. |
| Pathway | Pathway entry |
| DEGs with pathway annotation (2085) | Number of DEGs with pathway annotation |
| All genes with pathway annotation (8986) | Number of all genes with pathway annotation |
| Pvalue | P-value in Hypergeometric Test |
| Qvalue | Qvalue (Pathways with Qvalue≤0.05 are significantly enriched in DEGs) |
| Pathway ID | Pathway ID in KEGG |

Notice: pathways with Qvalue≤0.05 are significantly enriched in DEGs, see red-border region.

Through the enrichment analysis of DEGs pathway significance, we can get the pathways. Clicking the hyperlinks on pathways will take you to detailed pathway information in KEGG database.

**References**

1. 't Hoen, P. A., Y. Ariyurek, *et al*. (2008). "Deep sequencing-based expression analysis shows major advances in robustness, resolution and inter-lab portability over five microarray platforms." Nucleic Acids Res 36(21): e141.
2. Morrissy, A. S., R. D. Morin, *et al*. (2009). "Next-generation tag sequencing for cancer gene expression profiling." Genome Res.
3. Audic, S. and J. M. Claverie (1997). "The significance of digital gene expression profiles." Genome Res 7(10): 986-95.
4. Benjamini, Y. and D. Yekutieli (2001). "The control of the false discovery rate in multiple testing under dependency." The Annals of Statistics 29: 1165-1188.
5. Eisen, M. B., P. T. Spellman, *et al*. (1998). "Cluster analysis and display of genome-wide expression patterns." Proc Natl Acad Sci U S A 95(25): 14863-8.
6. Saldanha, A. J. (2004). "Java Treeview--extensible visualization of microarray data." Bioinformatics 20(17): 3246-8.
7. Kanehisa, M., M. Araki, *et al*. (2008). "KEGG for linking genomes to life and the environment." Nucleic Acids Res 36(Database issue): D480-4.
8. Hooper, S. D. and P. Bork (2005). "Medusa: a simple tool for interaction graph analysis." Bioinformatics 21(24): 4432-3.
